# Nano-modified viruses prime the tumor microenvironment and promote the photodynamic virotherapy in liver cancer

**DOI:** 10.1186/s12929-023-00989-1

**Published:** 2024-01-02

**Authors:** Da-Liang Ou, Zi-Xian Liao, Ivan M. Kempson, Lin Li, Pan-Chyr Yang, S.-Ja Tseng

**Affiliations:** 1 Graduate Institute of Oncology, National Taiwan University College of Medicine, Taipei, 10051 Taiwan; 2 National Taiwan University YongLin Institute of Health, National Taiwan University, Taipei, 10051 Taiwan; 3 Institute of Medical Science and Technology, National Sun Yat-Sen University, Kaohsiung, 80424 Taiwan; 4 Future Industries Institute, University of South Australia, Mawson Lakes, SA 5095 Australia; 5 Department of Internal Medicine, National Taiwan University College of Medicine, Taipei, 10051 Taiwan; 6 Institute of Biomedical Sciences, Academia Sinica, Taipei, 11529 Taiwan; 7 Program in Precision Health and Intelligent Medicine, Graduate School of Advanced Technology, National Taiwan University, Taipei, 10051 Taiwan

**Keywords:** Orthotopic hepatocellular carcinoma, Iron oxide nanoparticles, Adeno-associated virus, Immune-promoting effect, Macrophage, Photodynamic virotherapy

## Abstract

**Background:**

As of 2020, hepatocellular carcinoma (HCC), a form of liver cancer, stood as the third most prominent contributor to global cancer-related mortality. Combining immune checkpoint inhibitors (ICI) with other therapies has shown promising results for treating unresectable HCC, offering new opportunities. Recombinant adeno-associated viral type 2 (AAV2) virotherapy has been approved for clinical use but it efficacy is stifled through systemic administration. On the other hand, iron oxide nanoparticles (ION) can be cleared via the liver and enhance macrophage polarization, promoting infiltration of CD8^+^ T cells and creating a more favorable tumor microenvironment for immunotherapy.

**Methods:**

To enhance the efficacy of virotherapy and promote macrophage polarization towards the M1-type in the liver, ION-AAV2 were prepared through the coupling of ION-carboxyl and AAV2-amine using 1-ethyl-3-(3-dimethylaminopropyl)carbodiimide hydrochloride (EDC)/N-hydroxysulfosuccinimide (Sulfo-NHS). Efficacy after systemic delivery of ION-AAV2 in an orthotopic HCC model was evaluated.

**Results:**

After 28 days, the tumor weight in mice treated with ION-AAV2 was significantly reduced by 0.56-fold compared to the control group. The ION-AAV2 treatment led to an approximate 1.80-fold increase in the level of tumor associated M1-type macrophages, while the number of M2-type macrophages was reduced by 0.88-fold. Moreover, a proinflammatory response increased the population of tumor-infiltrating CD8^+^ T cells in the ION-AAV2 group. This transformation converted cold tumors into hot tumors.

**Conclusions:**

Our findings suggest that the conjugation of ION with AAV2 could be utilized in virotherapy while simultaneously exploiting macrophage-modulating cancer immunotherapies to effectively suppress HCC growth.

## Background

Liver cancer ranks as the third most common cause of cancer-related deaths worldwide [1, 2]. The predominant form of liver cancer is hepatocellular carcinoma (HCC), which constitutes approximately 90% of all liver cancer cases. Despite significant advancements in the strategies and combination therapies employed for HCC in the past decade, outcomes remain very poor with five year-survival rate being 64% [2, 3]. Immune checkpoint inhibitors (ICI) used clinically for advanced HCC treatment have approval by the US Food and Drug Administration (FDA) [4, 5]. However, ICI efficacy has not been considered satisfactory [6]. Moreover, only a small percentage of ICI interact with the target via systemic circulation due to off-target delivery [7, 8]. Combination therapies using ICI as a backbone represents the most noticeable advance of systemic therapy for unresectable HCC [9]. Therefore, it is attractive to develop new combined methods for advanced HCC.

Recently, cancer virotherapy has emerged as a promising clinical strategy. Adeno-associated virus (AAV) vectors stand as the forefront platform for delivering genes to treat a diverse range of human diseases [10, 11]. Notable achievements in preclinical and clinical applications of AAV-mediated gene expression, gene silencing, and gene editing have solidified AAV's reputation as a promising therapeutic vector [10, 11]. In the history of clinical trials, more than three thousand patients have been treated with AAV. AAV2 has been used in over 40% of clinical trials, particularly for liver-specific targeting [11]. Since 2017, three gene therapies utilizing recombinant AAV have gained approval [10, 12, 13]. However, the systemic administration of these therapies still presents significant challenges [14]. The majority of the vector still predominantly accumulates in the liver [15]. Simultaneously, the favorable characteristics of nanoparticles have been harnessed to enhance the efficacy of conventional cancer immunotherapy and introduce innovative strategies to combat cancer. For instance, iron oxide nanoparticles (ION)-conjugated with ICI [8] or AAV [16, 17] have promoted notable control of tumor growth in orthotopic HCC tumors or non-small cell lung cancers (NSCLC). Importantly, a multitude of preclinical and clinical trials have evaluated various ION formulations, with several achieving successful market entry [18]. The mechanistic contribution from the ION themselves is intriguing.

Tumor-infiltrating immune cells in the liver primarily consist of macrophages [19], with M1-type macrophages exerting oncolytic effects through pro-inflammatory responses. On the other hand, M2-type macrophages suppress immune responses while promoting angiogenesis and neovascularization. The negatively charged ION downregulate M2-associated arginase-1 in macrophages, leading to their polarization into M1-type macrophages by upregulating the interferon regulatory factor 5 signaling pathway [20]. In addition to their role in providing magnetic resonance imaging (MRI) contrast [17, 18], magnetic guidance [16, 18], and advantageous nanoformulation properties, ION offer the potential for synergistically reprogramming the tumor microenvironment, thereby greatly improving the outcomes of existing therapeutic approaches. Notably, ION-conjugated materials mainly accumulated in the liver, indicating a clear systemic circulation route [8, 16–18]. Like most nanoparticles administered intravenously, ION particles are eventually cleared by liver-residing macrophages via phagocytosis [21].

**Fig. 1:**
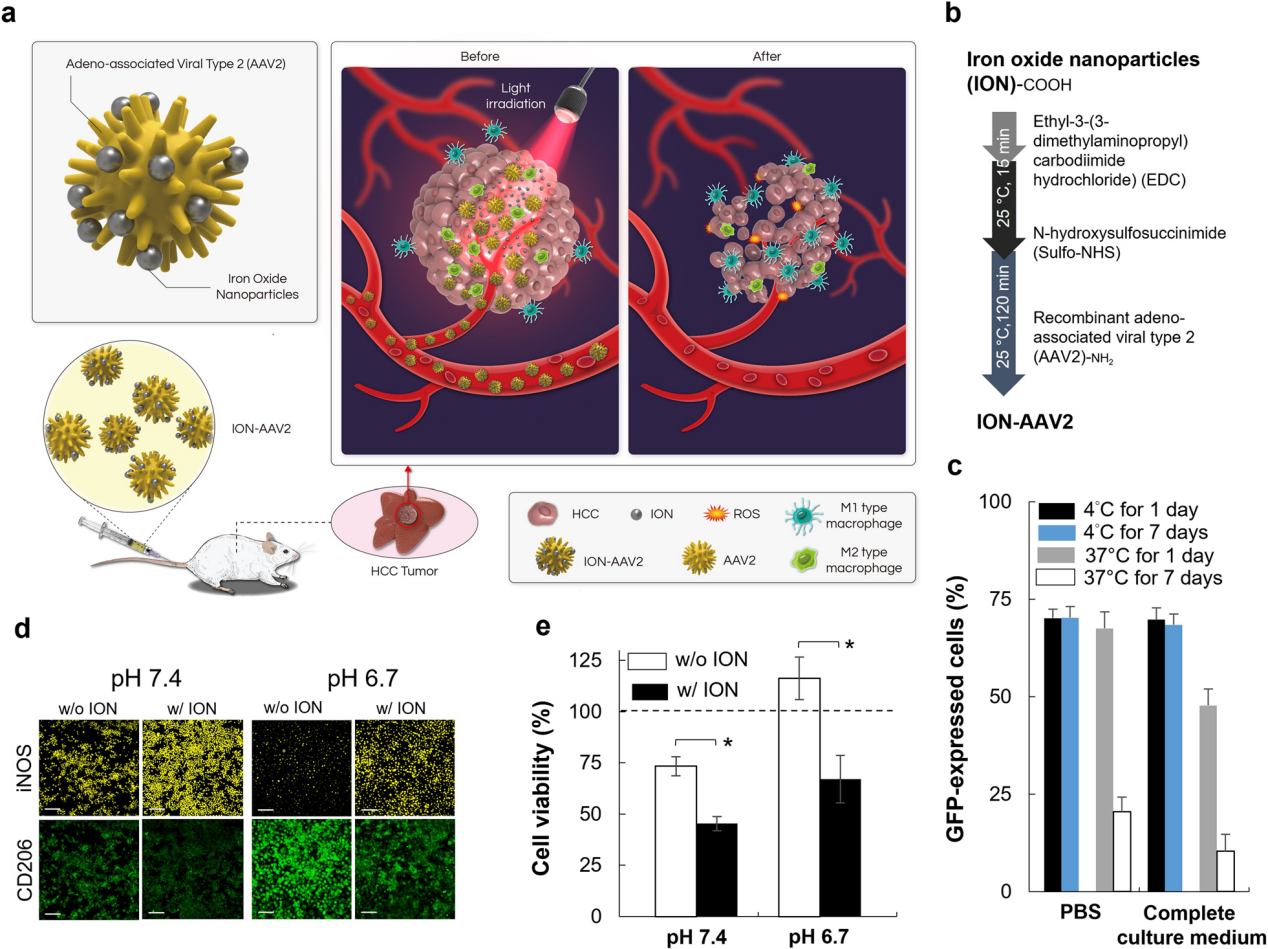
A nano-modified virus as a treatment for hepatocellular carcinoma (HCC). **a** A carrier with a liver-focused accumulation of iron oxide nanoparticles (ION)-conjugated recombinant adeno-associated viral type 2 (AAV2) concept involves the administration of a single tail vein injection to facilitate immune-promoting photodynamic virotherapy. Following systemic delivery, the tumor cells are infected with AAV2-KillerRed, leading to the expression of the KillerRed photosensitizer, which can be activated by light to initiate photodynamic virotherapy. Upon illumination, the KillerRed protein generates reactive oxygen species (ROS), causing intracellular damage and promoting cell death, ultimately resulting in a reduction in tumor size. Simultaneously, ION improves the ratio of M1/M2 type macrophages, enhancing their immunomodulatory function. **b** The conjugation of ION and AAV2 is schematically depicted, illustrating the process involving 1-ethyl-3-(3-dimethylaminopropyl)carbodiimide hydrochloride (EDC)/N-hydroxysulfosuccinimide (Sulfo-NHS) coupling. **c** The percentage of cells expressing GFP was assessed six days after transduction using Ironized AAV2 at a molar ratio of 1/20. This evaluation was conducted following incubation for either one day or seven days in either a PBS solution or a complete culture medium, at temperatures of 4 °C or 37 °C. Flow cytometry was employed for analysis, and the data presented represents the mean of measurements taken in six independent replicates, with the results reported as mean ± standard deviation. **d** Representative confocal images are shown, displaying RAW 264.7 M0 macrophages treated with ION under different conditions. Bar = 100 μm. **e** Cell viability of BNL-1MEA cells cocultured with treated macrophages incubated with ION at pH 7.4 or 6.7, as determined by MTS assays (*, *p* < 0.05, two-tailed unpaired Student’s t test). The bars represent the mean ± standard deviation (n = 4)

Anti-tumor M1-type macrophages contribute significantly to the infiltration of CD8^+^ T cells [19, 22] which contribute to the classification of tumors being ‘hot’ or ‘cold’ [23]. Cold tumors encompass immune-excluded and immune-desert tumor types. In immune-excluded tumors, CD8^+^ T cells are primarily confined to the invasion margins and struggle to efficiently penetrate the tumor. Beyond limited T-cell infiltration, cold tumors contain immunosuppressive cell populations, including tumor-associated macrophages (TAMs), T-regulatory cells (Tregs), and myeloid-derived suppressor cells (MDSCs). Conversely, immune-inflamed tumors, often referred to as 'hot tumors,' are distinguished by substantial infiltration of T-cells [23]. They are consequently one of the primary tumor-infiltrating immune cells for cancer immunotherapy delivery. Here, we hypothesized that a carrier with a liver-focused accumulation of ION, incorporated with an emerging virus enabled photodynamic virotherapy could promote therapeutic effects, remodeling HCC into hot tumor along with the highly specific sensitization of cancer cells to photo-irradiation. This hypothesis was tested using ION incorporated AAV2 (ION-AAV2) for transfecting to cells to express the phototherapeutic protein KillerRed after intravenous injection as conceptually depicted in Fig. 1a. KillerRed, a green fluorescent protein (GFP)-like dimer with low immunogenicity, incorporates a chromophore (Gln65-Tyr66-Gly67) within its β-barrel structure, featuring a unique water-filled channel. This chromophore efficiently absorbs green light in the 540–580 nm range, emitting longer-wavelength red light at 610 nm [16, 17, 24, 25]. This light-triggered process fosters the generation of reactive oxygen species (ROS) through oxygen and ion exchange with the surrounding environment, inducing potent phototoxicity. ION led to recruited and repolarization macrophages leading to a stimulation of CD8^+^ T cells. In combination with AAV2-KillerRed enabled photodynamic virotherapy, effective tumor remodeling and inhibition presented superior outcomes in terms of survival in an orthotopic mouse model.

## Methods

### Materials and cell culture

Phosphate buffered saline (PBS) was purchased from Sigma Co. (St. Louis, MO). 10 nm iron oxide nanoparticles (ION) with carboxylic acid (concentration: 4.3 nmole mL^−1^) was purchased from Ocean NanoTech (San Diego, CA, USA). (1-ethyl-3-(3-dimethylaminopropyl)carbodiimide hydrochloride) (EDC), 2-(N-morpholino)ethanesulfonic acid buffered saline (MESBS), and N-hydroxysulfosuccinimide (Sulfo-NHS) were purchased from Thermo Scientific Inc. (Rockford, IL, USA). Polyethyleneimine (branched, Mw = 25 K) was purchased from Aldrich (Milwaukee, MI). AAV2-Luciferase and AAV2-green fluorescent protein (GFP) were purchased from Cell Biolabs (San Diego, CA).

The cell lines used in this study were 293T cells (CRL-3216, ATCC), BNL-1MEA cells (TIB-75, ATCC), and mouse RAW 264.7 cells (TIB-71, ATCC), which were employed as M0 macrophages. These cells were cultured in Dulbecco's Modified Eagle's Medium (DMEM) supplemented with 100 μg mL^−1^ streptomycin, 10% fetal bovine serum (FBS), and 100 U mL^−1^ penicillin. The culture conditions involved maintaining the cells at a temperature of 37 °C with a 5% CO_2_ atmosphere.

### Standard process of viral production and purification

To generate AAV2-KillerRed, the AAV-2 Helper Free Packaging System (Cell Biolabs) was employed [16, 17, 25]. The production process involved co-transfection of plasmid DNAs (pHelper, pAAV-RC2, and pAAV-KillerRed) into 293T cells, using polyethyleneimine as the gene carrier. For each 100-mm dish, a combination of 20 μg of pHelper, 10 μg of pAAV-RC2, and pAAV-KillerRed was transfected into the 293T cells. The three plasmid DNAs were mixed with 40 μg of polyethyleneimine in serum-free culture medium, thoroughly vortex mixed for 30–60 s, and left for a minimum of 20–30 min. Transfection was carried out for only 30 min, after which the transfected cells were harvested three days later. The subsequent purification and titration of AAV2-KillerRed followed the protocols of the ViraBind™ AAV Purification Kit (Cell Biolabs) and QuickTiter™ AAV Quantitation Kit (Cell Biolabs) for viral transduction. The AAV2-KillerRed stock from each batch (eight 100-mm dishes) of virus production exhibited a range of 10^11^ to 10^12^ genome copies (GC) per milliliter. The purified viruses were then stored at − 80 °C until required.

### Synthesis, purification, and characterization of ION-AAV2

The synthesis of ION-AAV2, as outlined in the step-by-step procedures involving EDC/NHS coupling of ION-carboxyl and AAV2-amine (Fig. 1b), resulted in the complete formation of ION-AAV2 [16, 17]. To summarize, EDC (3.46 nmol) was gradually added to the ION with carboxylic acid (40 μL, 4.3 nmole mL^−1^) for molar ratios of ION/EDC at 1/20 in MESBS. The optimal molar ratio of ION:EDC in the ION-AAV2 using the EDC/NHS linker was identified as 1:20 based on prior reports [16, 17]. Subsequently, Sulfo-NHS was added over a period of 15 min to generate Sulfo-NHS ester-contained ION with amine reactivity. The AAV2 dose (5 × 10^8^ GC) was then introduced dropwise into the mixture and allowed to react for 120 min. Following the reaction, the ION-AAV2 solution was subjected to purification and solvent exchange using a desalting size-exclusion column (100 K molecular weight cutoff) with PBS as the exchange medium. Following the purification process, PCR utilizing the primers (5ʹ-GCCCATGAGCTGGAAGCC-3ʹ and 5ʹ-CGATGGCGCTGGTGATGC-3ʹ) yielded a recycling efficiency of approximately 90% for ION-AAV2-KillerRed [16, 17].

The quantity of iron linked to ION-AAV2 through chemical conjugation was ascertained using inductively coupled plasma atomic emission spectroscopy (ICP-AES). In this process, samples were subjected to a 24-h heating period to completely remove the medium. Following this, 1 mL of 37% HCl was introduced and mixed vigorously to ensure the full dissolution of iron oxide nanoparticles into an ionic state. The samples were then heated once again to 70 °C for 12 h to evaporate the HCl. Subsequently, the samples were combined with 3 mL of 2% nitric acid and filtered through a 0.2 μm surfactant-free cellulose acetate membrane filter (Thermo Scientific Inc.) to eliminate organic materials and impurities. ICP-AES analysis of the ION-AAV2 showed a yield of 52 ± 3.6 ION (with a total quantity of 25 μg) for molar ratios of ION/EDC at 1/20. All results are presented as a percentage of the initial iron concentration. The data represents the mean ± standard deviation from experiments conducted in triplicate.

To assess the stability of the Ironized virus, a 100 µL solution of ION-AAV2 (ION: 0.1725 nmol; AAV2: 5 × 10^8^ GC) with AAV2-GFP was mixed with 1 mL of PBS solution (pH 7.4) or complete culture medium (pH 7.4). The resulting mixture was then stored at either 4 °C or 37 °C for durations of 1 day or 7 days. Subsequently, the evaluation of the transduction capability of the ION-AAV2 was performed.

Cancer cells infected with ION-AAV2 carrying the KillerRed gene were subjected to green-light irradiation (540–560 nm) for a duration of 30 min, with a constant power level at the cellular level, measured at approximately 55 mW cm^−2^ using an optical-power meter from Thorlabs, Inc. (Newton, New Jersey, USA). Subsequent to irradiation, KillerRed exhibited the capacity to initiate the ROS generation upon exposure to visible light [16, 17, 24, 25]. The ROS production was induced by light was assessed in cell cultures by utilizing the CellROX Green reagent (Invitrogen) as a fluorescent marker.

### In vitro culture of macrophages with ION

In order to characterize M1-type or M2-type macrophages, we examined the expression of specific surface receptors, such as iNOS or CD206 [26]. RAW 264.7 M0 macrophages were seeded in each well of a 48-well plate at a density of 7 × 10^4^ cells, followed by an overnight culture in DMEM at 37 °C. Afterward, the RAW 264.7 M0 macrophages were incubated with or without ION (40 μL, 4.3 nmole mL^−1^) at pH 7.4 or 6.7 for a 3-day period. For the assessment of M1-type (iNOS^+^CD206^−^) or M2-type (iNOS^−^CD206^+^) macrophages, the ION-treated macrophages were fixed with 4% paraformaldehyde (PFA). Immunostaining was conducted using an anti-iNOS antibody to identify M1-type macrophages, and a mouse MMR/CD206 antibody to identify M2-type macrophages. Subsequently, immunofluorescence observation of iNOS and CD206 was performed using a donkey anti-rabbit IgG (H + L) highly cross-adsorbed secondary antibody, Alexa Fluor 555, and a donkey anti-goat IgG (H + L) cross-adsorbed secondary antibody, Alexa Fluor 488, respectively. Confocal microscopy was employed for the visualization of all macrophages.

Furthermore, it is worth noting that lipopolysaccharide (LPS) stimulates the expression of inducible nitric oxide synthase (iNOS) in M1 macrophages, resulting in an enhanced production of nitric oxide (NO) [26]. To investigate this phenomenon, a total of 9 × 10^4^ treated macrophages were co-cultured with 3 × 10^4^ BNL-1MEA cells in individual wells of a 24-well plate, followed by incubation in culture medium. Subsequently, the culture medium was supplemented with 100 ng mL^−1^ LPS and incubated for 24 h. The effects were assessed using the CellTiter 96® AQueous One Solution cell proliferation assay (Promega, Madison, WI). The untreated cells’ reduction of MTS was set at 100% and used as a reference, with the reduction in test cells expressed as a percentage relative to the untreated cells.

### Mice studies and assays

**Fig. 2:**
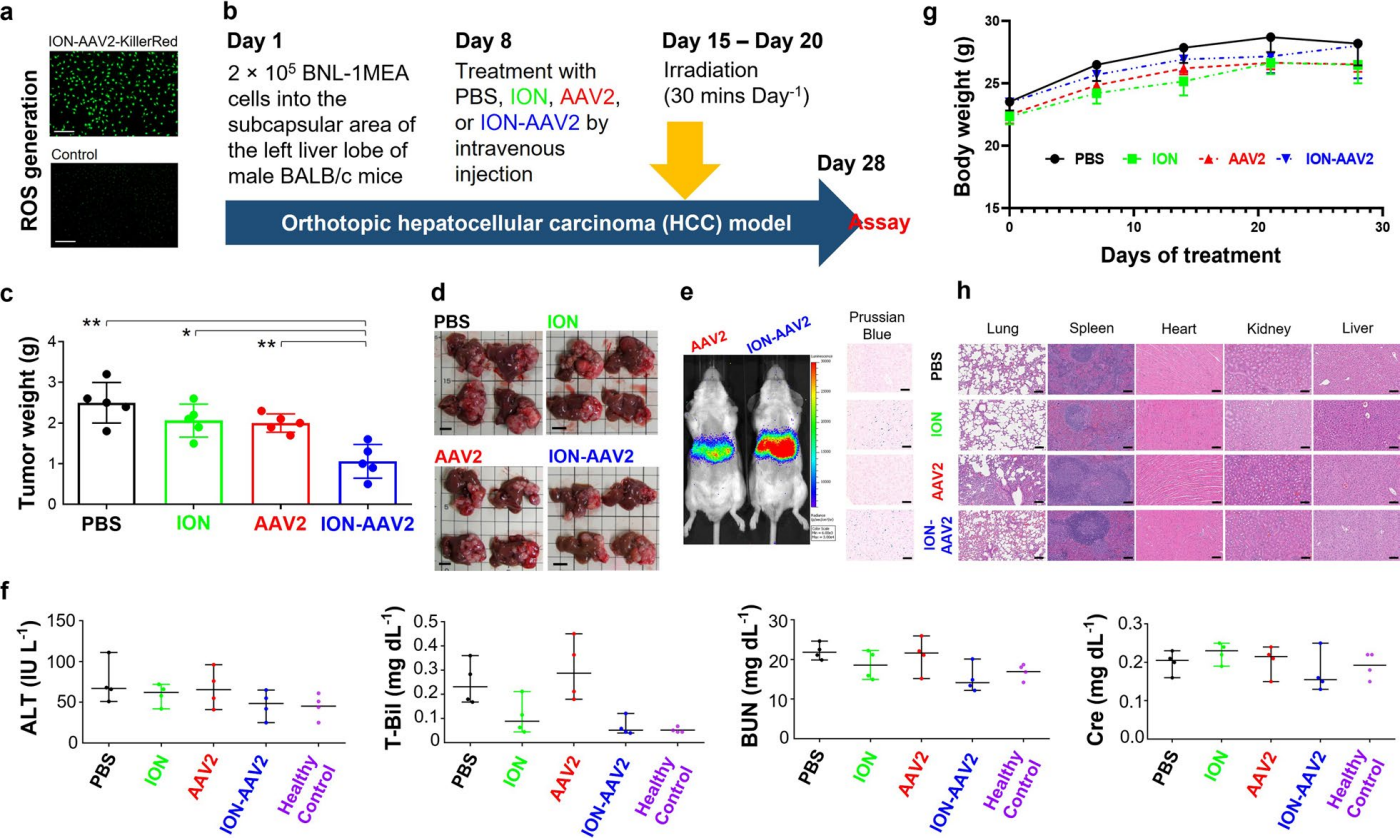
In vivo treatments using nano-modified virotherapy in the orthotopic model of HCC. **a** Photographs captured under designated light exposure durations showcasing ROS production resulting from the photoactivation of KillerRed within cancer cells. Bar = 200 μm. **b** Treatment protocol for mice with orthotopic BNL-1MEA HCC. **c** Tumor weight at Day 28 after various treatments (*, *p* < 0.05; **, *p* < 0.01, two-tailed unpaired Student’s t test). Bars represent the mean ± standard deviation (n = 5). **d** Representative images of orthotopic HCC tumors after various treatments are shown. Bar = 1 cm. **e** Photographs capturing representative IVIS images of mice acquired on Day 7 following the intravenous injection of different formulations, utilizing AAV2-encoded luciferase as a detection signal (left panel). Representative section images of liver stained by Prussian Blue after various treatments are displayed (right panel). Bar = 50 μm. **f** Blood biochemistry data 28 days after the indicated treatment. Data represent the mean ± standard deviation (n = 4). **g** Body weight of mice in response to various treatments. Data represent the mean ± standard deviation (n = 4). **h** Representative section images of lung, spleen, heart, kidney, and liver from mice after various treatments are displayed. Bar = 50 μm

The animal experiments conducted in this study were performed in compliance with the guidelines set forth in the "Guide for the Care and Use of Laboratory Animals”. Approval for the experiments was obtained from the Institutional Animal Care and Use Committee of the College of Medicine at National Taiwan University. Male BALB/c mice, aged 6–7 weeks, were procured from the National Laboratory Animal Center in Taiwan. We selected these mice based on their suitability for establishing an orthotopic model with BNL-1MEA cells, as they closely mimic the tumor microenvironment typically found in human liver cancer [8]. The model of orthotopic HCC was established using the implantation of ~ 2 × 10^5^ BNL-1MEA cells into the subcapsular area of the left liver lobe of mice and randomly assigned to different groups on Day 5 post-implantation of tumor. Treatment with PBS, ION (250 μg, 1.725 nmol), AAV2 (5 × 10^9^ GC mouse^−1^), or ION-AAV2 (ION: 1.725 nmol; AAV2: 5 × 10^9^ GC mouse^−1^) was given at Day 8 by intravenous injection via the retro-orbital sinus (Fig. 2a). For photodynamic therapy a yellow laser operating at a wavelength of 593 nm was employed and guided via an optical fiber, targeting the tumor surface with a beam spot measuring 6 mm in diameter. The laser fiber tip was oriented perpendicularly to the animal's body and positioned directly above the liver. At Day 15–Day 20, animals were locally treated with 1.5 mW mm^−2^ total irradiance (30 min day^−1^) for KillerRed activation. At Day 28, tumors were excised and measured. Major organs (liver, lung, spleen, heart, and kidney) were excised from mice from the different treatment groups and prepared for H&E analysis. Furthermore, liver sections underwent Prussian Blue staining to identify ION [8, 16, 17].

An injection of ION-AAV2-Luciferase (ION: 1.725 nmol; AAV2: 5 × 10^9^ GC mouse^−1^) or AAV2-Luciferase (5 × 10^9^ GC mouse^−1^), suspended in sterile-filtered PBS solution (with a total volume of 100 µL), was administered via the tail vein of mice. Bioluminescence imaging was conducted on Day 7 following the commencement of treatment. For this imaging procedure, mice were placed under anesthesia within a chamber filled with a 2% isoflurane and oxygen mixture. Luminescent images were captured 5 to 10 min after intraperitoneal injection of luciferin (approximately 240 µL, provided by Caliper Life Sciences Inc., Hopkinton, MA) using an IVIS imaging system (specifically, the Xenogen IVIS-50 equipped with Living Image software). The image acquisition time remained constant at 5 min, utilizing settings with a bin size of 16/4 and a field of view (FOV) of 12. In vivo bioluminescence signals were determined as the sum of both prone and supine acquisitions for each mouse, following background subtraction, and were expressed in units of photon flux per second per square centimeter per steradian (photon flux sec^−1^ cm^−2^ sr^−1^) originating from a region of interest encompassing the entire body.

To examine the distribution of immune cells within hepatocellular carcinoma (HCC), tumors were fixed in formalin, embedded in paraffin (FFPE), sectioned, and analyzed for infiltrating lymphocytes. The Polaris system from Akoya Biosciences, along with the Opal 7-Color Manual IHC Kit and anti-Rabbit Manual IHC Kit (Akoya Biosciences, NEL810001KT and NEL840001KT), were utilized for the detection of specific markers on immune cells in the FFPE sections. Opal620 was used to detect rabbit anti-CD4 (abcam), Opal570 for rabbit anti-CD8 (Bioss), Opal650 for rabbit anti-MHC II (Bioss), Opal520 for rabbit anti-CD19 (HistoSure) or rabbit anti-Foxp3 (R&D), Opal520 for rabbit anti-CD206 (Bioss), and Opal690 for rabbit anti-F4/80 (HistoSure) or rabbit anti-PD1 (Abcam). Multispectral imaging of each HCC tumor section was performed using the Phenochart and the inForm software (Akoya Biosciences). For the inForm software, the machine-learning mode was employed, wherein ten representative tumor images were selected as the training set. These images were manually annotated to identify the different cell types using specific markers: B cells (CD19^+^ stained), CD4^+^ T cells (CD4^+^ stained), CD8^+^ T cells (CD8^+^ stained), M1-type macrophages (F4/80^+^MHC II^+^CD206^−^ stained), M2-type macrophages (F4/80^+^MHC II^−^CD206^+^ stained), PD1^+^CD8^+^ T cells (PD1^+^CD8^+^ stained), and regulatory T cells (Treg cells, Foxp3^+^CD4^+^ stained). Cell segmentation was carried out based on nuclear staining using 4',6-diamidino-2-phenylindole (DAPI).

## Results

### ION-AAV2 stability

We presented the general form of the EDC/Sulfo-NHS modification of nano-modified virus where Sulfo-NHS ester ION react with amine-containing AAV2 to form ION-AAV2 (Fig. 1b). ION-AAV2 were assessed for their stability and by measuring their transduction effectiveness after storage periods of 1 day or 7 days in either PBS solution or a complete culture medium, and at either 4 °C or 37 °C. When stored at 4 °C, the activity remained consistent for a duration of 7 days in both media as evidenced by GFP expression in cells (Fig. 1c). However, a notable decline in transduction efficacy was observed following a 7-day storage period at 37 °C. This temperature-dependent degradation is likely attributed to factors such as thermal deterioration, serum protein coating, and potential aggregation or neutralization, as reported in previous studies [16, 27].

### ION modulates in vitro macrophage polarization

ION have recently been shown to improve the polarization of macrophages towards the M1-type [20]. The internalization of iron oxide nanoparticles enhances the conversion of M2-type to M1-type macrophages under neutral or acidic pH conditions [28]. Surprisingly, the negatively charged IONs can diminish the expression of arginase-1 associated with M2-type macrophages, thereby inducing their transformation into M1-type macrophages through the enhancement of the interferon regulatory factor 5 signaling pathway [20]. In order to investigate the impact of the ION used here on macrophage polarization, we examined the distribution of M1-type and M2-type macrophages derived from RAW 264.7 M0 macrophages treated with ION at different pH levels (7.4 or 6.7). An increase in the intensity of M1-type macrophages upon stimulation with ION incubation was observed (Fig. 1d). Furthermore, M2-type macrophages have been found to be more abundant in acidic tumor microenvironments [19, 26, 29]. Interestingly, we observed that ION treatment resulted in a significant reduction in the expression of CD206, a surface marker associated with M2-type macrophages, in RAW 264.7 M0 macrophages undergoing polarization and a notable increase in the expression of iNOS, a surface marker associated with M1-type macrophages [30]. These data show that ION incubation can modulate macrophage behavior, including their phenotype and polarization state [20]. When the BNL-1MEA cancer cells were co-cultured with macrophages and exposed to ION, a significant inhibition of cancer cell growth was observed (cell viability of 45% at pH 7.4; cell viability of 67% at pH 6.7), in contrast to BNL-1MEA cells that were not exposed to ION at pH 7.4 (cell viability of 73%) or pH 6.7 (cell viability of 116%) (Fig. 1e).

### Orthotopic HCC treated with photodynamic virotherapy

ION-AAV2 encoded KillerRed's light-induced mechanism stimulates the generation of robust phototoxicity, apoptosis, and anti-proliferation by producing ROS (Fig. 2a) [16, 17, 24]. Next, ION-AAV2 was evaluated in terms of inhibiting established orthotopic HCC tumor growth through photodynamic virotherapy combined with the altered immunosuppressive tumor microenvironment. In vivo efficacy of tumor suppression was assessed in the BALB/c model with an orthotopic BNL-1MEA comparing injection of PBS, ION, AAV2, or ION-AAV2 via the tail vein (Fig. 2b). Compared to PBS, treatment with ION or AAV2 alone yielded only slight tumor growth inhibition (Fig. 2c) while ION-AAV2 treated mice exhibited much smaller tumor (1.1 ± 0.416 g, compared to 2.5 ± 0.500 g for PBS, *p* = 0.0033; 2.1 ± 0.404 g for ION, *p* = 0.0326; 2.0 ± 0.224 g for AAV2, *p* = 0.0031) (Fig. 2c, d).

As expected, bioluminescence was observed in the liver, aligning with the predicted clearance pathways for AAV2 [15–17]. Notably, when contrasted with the administration of AAV2 alone, the use of ION-AAV2 resulted in a marked elevation in delivery to the liver, indicated by increased bioluminescence (left panel, Fig. 2e). The presence of ION-AAV2 stained by Prussian blue was consistently noted in the liver (right panel, Fig. 2e). Biochemical analyses, including alanine aminotransferase (ALT), blood urea nitrogen (BUN), total bilirubin (T-Bil), and creatinine (Cre) assays, were conducted to assess the effects of PBS, ION, AAV2, or ION-AAV2 treatment. No significant differences were observed among the groups (Fig. 2f). The weight of the mice remained relatively stable throughout the treatment, indicating minimal side-effects resulting from the administration of ION, AAV2, or ION-AAV2 (Fig. 2g). Hematoxylin–eosin staining (H&E staining) of lung, spleen, heart, kidney, and liver samples from the ION, AAV2, or ION-AAV2 treated group revealed minimal changes compared to the PBS control group (Fig. 2h). Overall, mice treated with ION-AAV2 did not exhibit significant deviations in blood test values or hemogram compared to the mice treated with PBS.

### In vivo distribution of immune cells in HCC tumor microenvironment

**Fig. 3:**
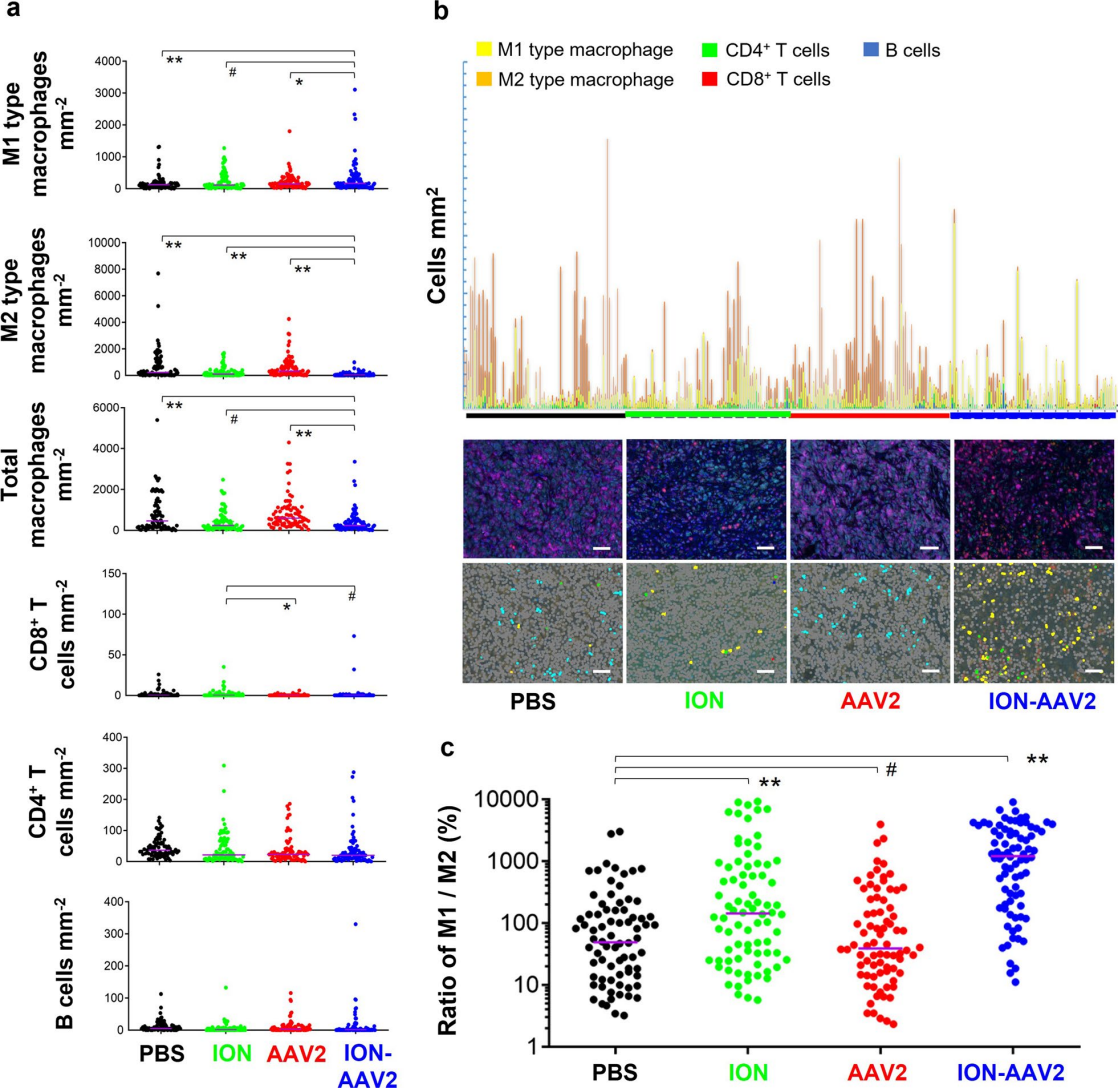
Immune cell distributions in mice after various treatments. **a** Measurment of tumor-infiltrating immune cells (B cell: CD19^+^ stained; CD4^+^ T cell: CD4^+^ stained; CD8^+^ T cell: CD8^+^ stained; M1 type macrophage: F4/80^+^MHC II^+^CD206^−^ stained; M2 type macrophage: F4/80^+^MHC II^−^CD206^+^ stained) in orthotopic HCC (*, *p* < 0.05; **, *p* < 0.01, ^#^ *p* > 0.05, two-tailed unpaired Student’s t test). Median with 95% confidence interval. **b** Tumor-infiltrating M1-type macrophages, M2-type macrophages, CD8^+^ T cells, CD4^+^ T cells, and B cells in formalin-fixed, paraffin-embedded tumor tissues were quantified using multiplex immunofluorescence staining after various treatments. Representative spectrally unmixed composite images (20 × magnification) are shown (yellow, M1-type macrophages; orange, M2-type macrophages; green, CD4^+^ T cells; red, CD8^+^ T cells; blue, B cells; grey, cell nucleus). Bar = 50 μm. **c** M1/M2-type macrophage ratio (**, *p* < 0.01; ^#^ *p* > 0.05, two-tailed unpaired Student’s t test) and imaging of the whole specimen area; each dot represents one acquisition field (80 fields / 5 mice tumor sections)

During the in vivo tumor growth experiments (Fig. 2c, d), we hypothesized that a portion of the antitumor effect exhibited by ION-AAV2 could be attributed to its ability to modify the immunosuppressive tumor microenvironment. To validate this hypothesis, tumor-infiltration of immune cells following various administrations as assessed. Within the tumor microenvironment, macrophages are among the most abundant immune cells, with M2-type macrophages being particularly prevalent [19]. On the other hand, M1-type macrophages play a crucial role in facilitating the activation of antitumor inflammation, such as by promoting increased interferon (IFN)-γ production by T cells [14]. ION, AAV2, and ION-AAV2 all promoted M1-type macrophages (F4/80^+^MHC II^+^CD206^−^ stained) compared to PBS (Fig. 3a), being ~ 124%, 122%, or 188% greater respectively. Simultaneously, M2-type macrophages (F4/80^+^MHC II^−^CD206^+^ stained) significantly decreased in the ION (38.5%, compared to PBS) and ION-AAV2 (11.6%, compared to PBS) treated groups. AAV2 treatment alone however did not exhibit an ability to significantly impact this population, highlighting the importance of the ION. Furthermore, our observations revealed a decrease in the overall macrophage count in response to both ION and ION-AAV2.

Activated M1-type macrophages typically mediate cytotoxic tumor killing and phagocytosis of cancer cells. Simultaneously, they’re also linked with immunomodulation orchestrated by CD8^+^ T lymphocytes and interferons [19, 22]. Furthermore, the CD8^+^ T cells can destroy virus-infected cells and cancer cells, and produce proinflammatory cytokines such as IFN-γ [31]. As M1-type macrophages promote T cell recruitment it was anticipated that the tumor-infiltrating CD8^+^ T cells (CD8^+^ stained) population also increased for the ION and ION-AAV2 groups (Fig. 3a), representing an inflamed tumor immune phenotype. Intriguingly, all treatments had non-significant effect on recruitment of CD4^+^ T cells (CD4^+^ stained) and B cells (CD19^+^ stained). Taken together, the in vivo experiments revealed that ION or ION-AAV2 activated the responses of CD8^+^ T cells, presumably via inducing M1 macrophage recruitment. The fluorescent imaging of M1-type macrophages, M2-type macrophages, CD8^+^ T cells, CD4^+^ T cells, or B cells (Fig. 3b) supports the data in Fig. 3a.

Treatment with ION and ION-AAV2 demonstrated a substantial increase in the M1/M2-type macrophage ratio compared to PBS (*p* = 0.0015 for ION; *p* = 4.55 × 10^–12^ for ION-AAV2, Fig. 3c), which is consistent with the findings in Fig. 3a and b. These results provide clear evidence that ION-AAV2 possesses immunomodulatory properties that effectively recruit M1-type macrophages into the tumor microenvironment. This observation aligns with the improved control of HCC tumors when combined with photodynamic therapy, as depicted in Fig. 2c and d.

**Fig. 4:**
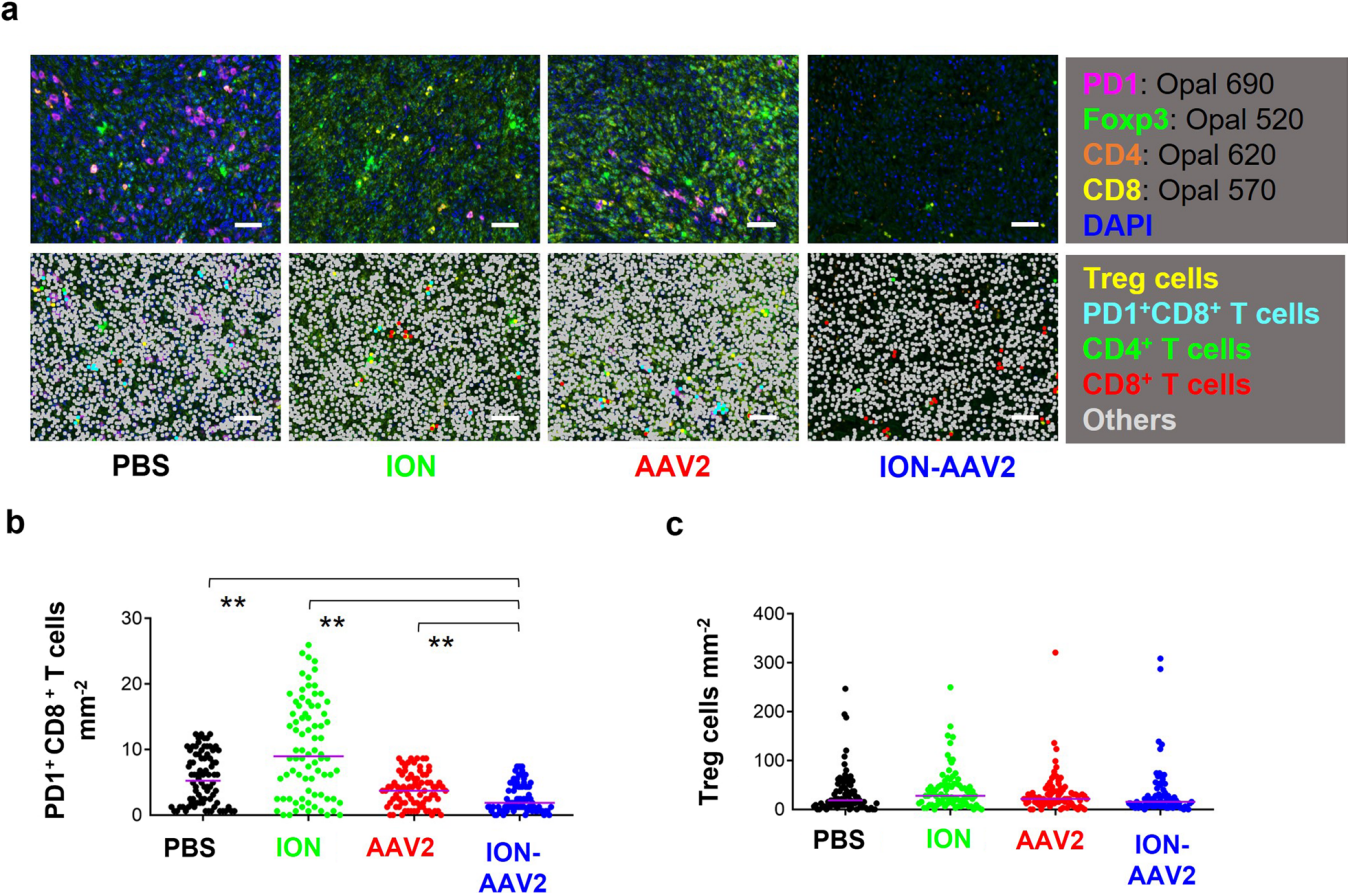
Immunosuppressive cell distributions in mice after various treatments. **a** Assessment of tumor-infiltrating exhausted PD1^+^CD8^+^ T cells and Treg cells in formalin-fixed, paraffin-embedded tumor tissues using multiplex immunofluorescence staining after various treatments. Representative spectrally unmixed composite images (20 × magnification) are shown. Bar = 50 μm. **b** and **c** Quantification of tumor-infiltrating immunosuppressive cells (Exhausted PD1^+^CD8^+^ T cell: PD1^+^CD8^+^ stained; Treg cell: Foxp3^+^CD4^+^ stained; CD4^+^ T cell: CD4^+^ stained; CD8^+^ T cell: CD8^+^ stained) in orthotopic HCC (**, *p* < 0.01, two-tailed unpaired Student’s t test). Median with 95% confidence interval. Each dot represents one acquisition field (80 fields / 5 mice tumor sections)

Furthermore, we conducted an analysis of immunosuppressive cell populations, with a specific focus on Treg cells (CD4^+^Foxp3^+^ stained) and exhausted PD1^+^CD8^+^ T cells (PD1^+^CD8^+^ stained), utilizing multiplex immunofluorescence staining within the tumor microenvironments (Fig. 4a). Notably, the exhausted PD1^+^CD8^+^ T cell population exhibited a significant reduction in the ION-AAV2 group, while there were no discernible differences in the staining results for Treg cells (Fig. 4b). These findings lend support to our hypothesis that ION-AAV2's immunomodulatory properties effectively mitigate the presence of exhausted PD1^+^CD8^+^ T cells within the tumor microenvironment.

## Discussion

Treating HCC with immunotherapy is challenging due to its diverse etiology and the presence of an immunosuppressive tumor microenvironment [3–5]. Developing efficient targeted delivery methods and exploring combination therapies remain primary opportunities in HCC research. AAV vectors have risen to prominence as a promising platform for delivering genes in response to a wide range of human diseases. Recent advances in developing clinically advantageous AAV capsids, optimizing genome designs, and harnessing groundbreaking biotechnologies have substantially driven the growth of the gene therapy field [10, 11]. Remarkable accomplishments in preclinical and clinical utilization of AAV have firmly established AAV as effective therapeutic vectors [10, 11]. This recognition is further emphasized by the regulatory approval of two AAV-based therapies in Europe and the United States [10, 12, 13]. In principle AAV2 promote dose escalation for systemic delivery, however the majority of the vector still ultimately accumulates in the liver [15] and current formulations lack control over in situ release and subsequent infection of target cells for effective virotherapy [10, 13]. The need for localized irradiation of infected cells to induce a therapeutic response presents an opportunity to avoid off-target toxicities [16, 17, 24, 25]. Notably, evaluating the presence of anti-AAV antibodies in patients before the administration of systemic gene therapy is a crucial factor in assessing the therapy's effectiveness and safety [32]. In preceding findings [8, 16, 17], ION-AAV2 primarily gathered in the liver, demonstrating a well-defined pathway for systemic circulation. Similar to most other nanoparticles, when ION is injected intravenously, they are ultimately eliminated by macrophages located in the liver, spleen, lymph nodes, and bone marrow, i.e., the mononuclear phagocytic system (MPS) [21]. Owing to their notable affinity for the liver, IONs have found widespread application in the visualization of primary liver abnormalities, such as HCC, and liver metastases [18]. Our in vivo studies demonstrate that ION-conjugated viruses accumulate in the liver, improving the efficacy of localized virotherapy [16, 17]

Among various nanoparticles, ION have shown particular promise in assisting cancer immunotherapy [20, 33, 34]. Negatively charged ION mediate the polarization of M1-type macrophages through the interferon regulatory factor 5 signaling pathway [20]. Infiltration of CD8 + T cells, which promotes tumor control, can be further enhanced by the immuno-stimulatory cytokines produced by M1-type macrophages [19, 22, 31]. Conversely, M2-type macrophages hinder outcomes by reducing the migration and invasion of CD8^+^ T cells into tumors [22]. Depleting tumor-associated M2-type macrophages restores CD8^+^ T cell activity. Our findings support these observations, as ION and ION-AAV2 promote M1-type macrophages in orthotopic HCC and are associated with the recruitment of CD8^+^ T cells. However, it is important to note that an increase in M1-type macrophages or CD8^+^ T cells alone is insufficient to promote tumor regression. When examining AAV2 within the context of ION-AAV2, the AAV2-mediated expression of KillerRed demonstrates the capacity to be directly initiated within cells. When appropriately exposed to light, KillerRed can efficiently initiate the generation of reactive oxygen species (ROS), resulting in cell death [16, 17, 24, 25]. This unique attribute of KillerRed proves valuable for activating light-sensitive proteins, selectively targeting specific cell populations within living organisms, and exploring intracellular phenomena. On the other hand, numerous lines of evidence support the involvement of ROS in the detection of danger signals, encompassing the presence of pathogens and tissue damage. Specifically, Pathogen-Associated Molecular Pattern (PAMP) and Damage-Associated Molecular Pattern (DAMP) recognition by immune cells can instigate intracellular signaling cascades that lead to heightened ROS production, potentially resulting in inflammasome activation and the production of pro-inflammatory cytokines [35, 36]. Furthermore, ROS assume a critical regulatory role in shaping the initiation and outcome of phagocytosis. They participate in the recognition and engulfment of compromised cells [37], while phagocytic cells like monocytes, macrophages, and neutrophils generate ROS during the oxidative burst, which is essential for pathogen eradication and the clearance of damaged cells [38]. Overall, the data indicate ION-AAV2 activate M1-type macrophages and T cells. In combination with the AAV2-activated photodynamic virotherapy improved outcomes in terms of tumor growth inhibition are achieved.

Expanding on our photodynamic virotherapy platform, we have successfully shown that the nano-engineered virus (ION-AAV2) exhibits a significant ability to stimulate immunological modulation within the tumor microenvironment. Reprogramming populations of the M1/M2-type macrophages specifically inhibit the progression of various solid tumors [8, 19, 39]. Efforts should be focused on developing ION to further enhance and optimize formulation parameters, enabling greater promotion of the M1-type macrophage population and enhancing combinatorial therapeutic strategies.

Numerous unresolved issues surround the mechanistic regulation and functional outcomes associated with macrophage activation and polarization, necessitating further investigation. Firstly, this study has delineated macrophage polarization in the context of ION-AAV2 and its effects on anti-tumor immunity. It's important to note that the activation of tumor-associated macrophages involves a complex interplay of diverse and dynamic proinflammatory and anti-inflammatory signals within the tumor microenvironment. As a result, our present study may not distinguish between the elevation of M1 macrophages due to infiltration or the repolarization of existing tumor-associated M2 macrophages [40]. While it is conceivable that ION-AAV2 directly stimulates anti-cancer phenotypes in macrophages and lymphocytes, other mechanisms might also be in play. It's plausible that ION-AAV2 initially enhances the immunogenicity of cancer cells, subsequently leading to the reprogramming of macrophages into an anti-cancer phenotype. Typically, lymphocyte activation relies on antigen-presenting cells (APCs) such as dendritic cells and macrophages [41]. Furthermore, the simplistic dichotomous classification of macrophage activation as M1 or M2 may oversimplify a complex reality. Advancements in high-resolution analysis techniques for macrophage phenotypes hold the potential to shed light on how TAMs are influenced by their ontogeny and tissue-specific stress signals, which in turn impact their activation and function [42].

## Conclusion

Employing chemical modification to attach AAV2 to ION facilitates its efficient systemic delivery to orthotopic HCC tumors. This innovative approach leads to the buildup of AAV2 within the liver, resulting in a marked reduction in HCC tumor size. Significantly, the inclusion of ION in the formulation confers substantial advantages by counteracting the immunosuppressive microenvironment within HCC. It accomplishes this by recruiting M1-type macrophages and activating CD8 + T cells, thereby enhancing the therapeutic efficacy of the treatment.

## Data Availability

The datasets used and analyzed in this study are available from the corresponding author upon reasonable request.
